# Predictors of mortality of pediatric cancer patients admitted to the intensive care unit in a low-middle-income country

**DOI:** 10.3389/fped.2026.1720257

**Published:** 2026-02-24

**Authors:** Rana Helmy, Reham Khedr, Youssef Madney, Mohamed Kamal, Mark W. Kieran, Ali Mostafa, Alaa Elhaddad

**Affiliations:** 1 Department of Pediatric Oncology, Children’s Cancer Hospital, Cairo, Egypt; 2 Department of Pediatric Oncology, National Cancer Institute, Cairo University, Cairo, Egypt; 3 Department of Research and Biostatistics, Children’s Cancer Hospital, Cairo, Egypt; 4 Department of Pediatrics, Cairo University, Cairo, Egypt

**Keywords:** pediatric oncology, ICU admissions, mortality predictors, respiratory failure, septic shock, LMICs, hematological malignancies, acute lymphoblastic leukemia

## Abstract

**Background:**

Advancements in cancer therapies have markedly increased survival rates among patients. However, this progress has also led to a growing number of pediatric cancer patients requiring admission to intensive care units due to the severity of their disease and complications arising from treatment. It is essential to identify the predictors of mortality within this population to enhance clinical outcomes effectively.

**Methods:**

A retrospective study included patients younger than or aged 18 years old at diagnosis of malignancy who were admitted to the medical ICU in The Children Cancer Hospital, Egypt, from January 1, 2019, to August 1, 2021. The primary objectives were to determine the mortality rate, identify the common causes of ICU admissions, and analyze the predictors of mortality among the pediatric cancer patients admitted to the ICU.

**Results:**

A total of 1,501 ICU admissions were included. The most common causes of admission were sepsis (39%) and respiratory failure (31%). The mortality rate for the whole cohort was 32%. The most common causes of death were sepsis (46%) and disease progression/relapse (28.6%). Multivariable analysis identified higher mortality for patients admitted with septic shock (OR =  6.01, 95%CI 3.97–9.23, *P* < 0.001) and respiratory failure (OR =  6.35, 95%CI 4.13–9.97, *P* < 0.001), patients with progressive disease (OR =  1.86, 95%CI 1.38–2.50, *P* < 0.001), those transferred from inpatient wards (OR =  1.63, 95%CI 1.20–2.22, *P* = 0.002) and patients with longer ICU stay (OR =  1.04, 95%CI 1.04–1.06, *P* < 0.001).

**Conclusion:**

Pediatric cancer patients admitted to the ICU in LMICs have a high mortality rate, which confirms the need for targeted strategies to improve outcomes in this vulnerable population. Key approaches suggested by our research include early cancer diagnosis, optimized identification of early warning signs of critical illness, and decreasing sepsis-related mortalities by strict infection control, early diagnosis, and antimicrobial stewardship programs.

## Introduction

Advances in the field of oncology, including the introduction of intensified multimodality treatment protocols, better stratification, and advanced supportive care, have led to a substantial improvement in survival rates for patients with cancer. However, this has also increased the number of pediatric cancer patients requiring admission to the intensive care unit (ICU) due to the severity of their illness and treatment-related complications (1–3).

While PICU mortality rates have steadily declined for children without cancer, dropping from 11% in 1982 to just 2.7% by 2015, the mortality rates for pediatric cancer patients requiring PICU admission have remained persistently high. In 2019, the mortality rate among pediatric cancer patients in the PICU, excluding postoperative cases, was nearly 20%—a figure that has remained largely unchanged over time (4, 5).

Recent studies showed that about 40% of all pediatric cancer patients require admission to the pediatric intensive care unit (PICU) at some point during their disease course, with acute respiratory failure and sepsis being the main admission reasons (5).

In low-middle-income countries (LMICs), where healthcare resources may be more limited, understanding the factors that predict mortality among these critically ill pediatric cancer patients is crucial for improving patient outcomes. Existing studies have identified several factors associated with poorer outcomes among pediatric cancer patients admitted to the intensive care unit (ICU). Patients with hematological malignancies, a history of bone marrow transplantation, those who develop cardiovascular, respiratory, or renal failure, and those who required inotropic support tend to have worse prognoses (6–8). Conversely, patients admitted to the ICU at the time of initial cancer diagnosis appear to have better outcomes compared to those admitted during active treatment (8, 9).

In this context, it is essential to identify reliable prognostic factors that can guide clinicians in the early recognition of pediatric cancer patients who may benefit from timely ICU admission and appropriate management that will eventually lead to improving the survival of this vulnerable patient population.

The Children Cancer Hospital Egypt (CCHE-57357) is a leading tertiary pediatric oncology center in a low-to-middle income country (LMIC) setting with over 500 intensive care unit (ICU) admissions annually. In this context, we aim to collect and analyze data on the primary causes and the mortality rate of ICU admissions, as well as the key predictors of ICU mortality among our patient population. The overarching goal is to identify the critical interventions that need to be implemented to improve outcomes for these critically ill pediatric cancer patients within the LMIC healthcare environment. Insights gained from this institutional experience can inform the development of tailored protocols and resource allocation strategies to optimize the management and survival of this vulnerable patient group.

### Patients and methods

This retrospective study included patients aged 18 years or younger at diagnosis of a malignancy and who were admitted to the medical ICU at The Children Cancer Hospital Egypt (CCHE-57357) from January 1, 2019, to August 1, 2021. Elective perioperative admissions were excluded, as these patients are typically admitted to the surgical ICU.

The primary objectives of this study were to determine the mortality rate of pediatric cancer patients admitted to the ICU, identify the common causes of ICU admissions, and assess the predictors of mortality. Our secondary objective was to ascertain the attributable mortality rate. The study received approval from the Institutional Ethics Committee at our institution, which waived the need for informed consent due to the retrospective nature of this study.

Following a review of the literature to identify common predictors of mortality in cancer patients, we screened the computerized electronic medical records and laboratory records of patients meeting the eligibility criteria. Data collected included demographics, disease type (hematological, solid malignancy including CNS tumors, or post-hematopoietic stem cell transplant), remission status (new case diagnosed but not yet treated, remission achieved, under treatment but not yet evaluated, or progressive disease), transfer status from the inpatient ward or emergency room, length of inpatient stay before ICU admission, length of ICU admission, cause of ICU admission (type of organ dysfunction at ICU admission), presence of septic shock, presence of oncological emergencies (such as tumor lysis, hyperleukocytosis, or superior mediastinal syndrome), and ICU interventions, including the need for inotropic support, hemodialysis, and mechanical ventilation. Causes for ICU admission were recorded based on the main symptoms at the time of admission, with definitions of septic shock and organ dysfunctions established according to the International Pediatric Sepsis Consensus Conference (10).

### Statistical methods

Data were analyzed using R version 4.2. Descriptive statistics were used to summarize the baseline demographic, clinical, and ICU-related variables. Categorical data were presented as frequencies and percentages (*n*, %). All numerical variables were summarized using medians and interquartile ranges (IQR). Univariate comparisons between ICU survivors and non-survivors were performed using Pearson's Chi-squared test or Fisher's exact test for categorical variables, as appropriate. The Wilcoxon rank-sum (Mann–Whitney *U*) test was used to compare continuous variables between the two outcome groups. To identify independent predictors associated with ICU mortality, a multivariable logistic regression model was developed. We used backward elimination guided by the Bayesian Information Criterion to construct the multivariable logistic regression model. Results from the logistic regression analysis are presented as Odds Ratios (OR) with corresponding 95% Confidence Intervals (CI). A *p*-value of less than 0.05 was considered statistically significant throughout the analyses. Sample size calculations were performed *a priori* using established methods for multivariable prediction model development to ensure adequate statistical power (21). A nomogram was subsequently developed based on the final multivariable model to provide a graphical tool for risk estimation.

## Results

Over the study period, 1501 ICU admissions were included. The median age at ICU admission was 8.0 years (IQR 4.0, 13.0), and the majority of patients were male (57%). Regarding disease characteristics, hematological malignancies were the most common underlying diagnosis, accounting for 55% of cases, followed by solid tumors at 42%. A small percentage of patients (2.3%) were admitted post-hematopoietic stem cell transplantation, primarily due to the lower number of transplant patients relative to the overall hospital population, which they represent at approximately 4%. The five most frequent specific diagnoses were Acute Lymphoblastic Leukemia (24%), Brain Tumors (18%), Acute Myeloid Leukemia (13%), Non-Hodgkin's Lymphoma (12%), and Neuroblastoma (9.7%). At the time of ICU admission, almost half of the patients were in remission (45%), while 20% had progressive disease, 18% were classified as not yet evaluated, and 17% were newly diagnosed prior to cancer treatment.

Most patients were transferred to the ICU from inpatient wards (59%), with the remainder admitted directly from the emergency room (41%). The median length of hospital stay before ICU transfer was 2 days (IQR 0, 17), and the median length of stay within the ICU was 4 days (IQR 2, 8).

At ICU admission, various organ dysfunctions were prevalent. Cardiovascular dysfunction was reported in 40% of admissions, of which 21% were admitted with septic shock. Gram-negative organisms were the predominant type identified, accounting for 94% of septic shock cases. The remaining 19% were admitted with cardiovascular compromise without septicemia. Respiratory failure occurred in 25.9% of admissions. Cerebral failure was noted in 22% of admissions presenting as status epilepticus or altered consciousness. Disease-related oncologic emergencies such as Tumor Lysis Syndrome (8.1%), Superior mediastinal syndrome (1.5%), and Hyperleukocytosis (1.0%) were also noted.

Common ICU interventions included mechanical ventilation (48%), inotropic support (40%), and renal replacement therapy (7.2%). The overall ICU mortality rate was 32%, with 477 patients dying during their ICU admission (Table 1).

**Table 1:** Baseline demographic, clinical, and ICU characteristics of pediatric oncology patients.

Classifications	Characteristic	Category/Level	Value (*N* = 1,501)
Demographics	Age (years)	Median [IQR]	8.0 [4.0, 13.0]
	Sex	Female	639 (43%)
		Male	862 (57%)
Disease Characteristics	Malignancy Type	Hematological (H)	830 (55%)
		Solid Tumor	637 (42%)
		Post-HSCT (BMT)	34 (2.3%)
	Disease Status at ICU Admission	New Diagnosis	248 (17%)
		In Remission	682 (45%)
		Under Treatment/Not yet evaluated	274 (18%)
		Progressive Disease	297 (20%)
Admission & Stay Details	Transfer Source	Emergency Room (ER)	621 (41%)
		Inpatient Ward (IP)	879 (59%)
		Unknown	1 (<0.1%)
	Hospital Stay Before ICU (days)	Median [IQR]	2 [0, 17]
	ICU Length of Stay (days)	Median [IQR]	4 [2, 8]
Organ Dysfunction at ICU Admission	Respiratory Failure	Yes	388 (25.9%)
	Cardiovascular (CVS) Dysfunction	Yes	599 (40%)
		With Septic Shock	310 (21%)
	If Septic Shock, Organism Type:	Fungal	5 (1.6% of Sepsis)
		Gram-negative	291 (94% of Sepsis)
		Gram-positive	14 (4.5% of Sepsis)
	Cerebral Failure	Yes	333 (22%)
	Renal Dysfunction	Yes	77 (5.1%)
	Hepatic Dysfunction	Yes	18 (1.2%)
	Electrolyte Disturbance	Yes	193 (13%)
	Multi-Organ Dysfunction (MODS)	Yes	113 (7.5%)
	Disease-Related Emergency	Tumor Lysis Syndrome (TLS)	121 (8.1%)
		Hyperleukocytosis	15 (1.0%)
		Superior Mediastinal Syndrome (SMS)	23 (1.5%)
ICU Interventions	Inotropic Support	Yes	599 (40%)
	Mechanical Ventilation	Yes	717 (48%)
	Renal Replacement Therapy (RRT)	Yes	108 (7.2%)
Outcome	ICU Mortality	Dead	477 (32%)
		Alive	1,024 (68%)

*N,* number of ICU admissions. Data are presented as *n* (%) or Median [Interquartile range, IQR]. RRT, received dialysis. Cerebral failure = convulsions (seizures) and/or depressed level of consciousness.

A univariate analysis was performed to compare demographic, clinical, and ICU characteristics between patients who survived their ICU stay (*N* = 1024) and those who did not (*N* = 477). Significant differences were observed across numerous factors (*p* < .05). Patients who died in the ICU were younger than survivors, with a median age of 7.0 years compared to 8.0 years (*p* = .002). Non-survivors were significantly more likely to have an underlying hematological malignancy (60% vs. 53%, *p* = .023). Disease status at admission showed marked differences (*p* < .001); non-survivors were significantly more likely to have progressive disease (28% vs. 16%) or be classified as having not yet evaluated (23% vs. 16%), and less likely to be newly diagnosed (11% vs. 19%) or in remission (38% vs. 49%).

Regarding admission and stay details, non-survivors were significantly more likely to be transferred from an inpatient ward (75% vs. 51%, *p* < .001) and had significantly longer median lengths of stay both in the hospital before ICU admission (10 days vs. 1 day, *p* < .001) and within the ICU itself (6 days vs. 4 days, *p* < .001).

The prevalence of organ dysfunction at ICU admission was significantly higher among non-survivors. Notably, septic shock (*p* < .001), respiratory failure (*p* < .001), electrolyte disturbance (*p* < .001), cerebral failure (*p* < .001), hepatic dysfunction (*p* = .001), cardiovascular dysfunction (*p* < .001), and multi-organ dysfunction syndrome (*p* < .001) were all significantly more common in the non-survivor group.

The need for intensive interventions was significantly greater among non-survivors, including mechanical ventilation (97% vs. 25%, *p* < .001) and inotropic support (86% vs. 18%, *p* < .001). The requirement for renal replacement therapy was slightly higher in non-survivors (8.0% vs. 6.8%), yet this difference was not statistically significant (*p* = .4) (Table 2).

**Table 2:** Univariate comparison of characteristics between pediatric oncology ICU survivors and non-survivors.

Classifications	Characteristic	Category/Level	Alive (*N* = 1,024)	Dead (*N* = 477)	*p*-value
Demographics	Age (years)^a^	Median [IQR]	8.0 [4.0, 13.0]	7.0 [3.0, 12.0]	0.002**
	Sex^b^	Female	430 (42%)	209 (44%)	0.500
		Male	594 (58%)	268 (56%)	
Disease Characteristics	Malignancy Type^b^	Hematological (H)	543 (53%)	287 (60%)	0.023*
		Solid Tumor	459 (45%)	178 (37%)	
		Post-HSCT (BMT)	22 (2.1%)	12 (2.5%)	
	Disease Status at ICU Admission^b^	New Diagnosis	195 (19%)	53 (11%)	<.001*
		In Remission	500 (49%)	182 (38%)	
		No Yet Evaluated	166 (16%)	108 (23%)	
		Progressive Disease	163 (16%)	134 (28%)	
Admission & Stay Details	Transfer Source^b^	Emergency Room	501 (49%)	120 (25%)	<.001*
		Inpatient Ward (IP)	522 (51%)	357 (75%)	
		Unknown	1 (<0.1%)	0 (0%)	
	Hospital Stay Before ICU (days)^a^	Median [IQR]	1 [0, 11]	10 [0, 25]	<.001*
	ICU Length of Stay (days)^a^	Median [IQR]	4 [2, 7]	6 [2, 12]	<.001*
Cause of ICU Admission	Cardiovascular (CVS) Dysfunction^b^	Yes	338 (33%)	261 (55%)	<.001*
		No	686 (67%)	216 (45%)	
	Septic Shock	No Septic Shock	902 (88%)	289 (61%)	<.001*
	If Yes, Organism Type^b^	Fungal	3 (0.3%)	2 (0.4%)	
		Gram-positive	4 (0.4%)	10 (2.1%)	
		Gram-negative	115 (11%)	176 (37%)	
	Respiratory Failure	No Respiratory Failure	786 (77%)	327 (69%)	<.001*
	If Yes, Presumed Cause^b^	Infectious	73 (7.1%)	49 (10%)	
		Non-infectious	135 (13%)	59 (12%)	
		Undetermined cause	30 (2.9%)	42 (8.8%)	
	Electrolyte Disturbance^b^	Yes	183 (18%)	10 (2.1%)	<.001*
		No	841 (82%)	467 (98%)	
	Cerebral Failure^b^	Yes	256 (25%)	77 (16%)	<.001*
		No	768 (75%)	400 (84%)	
	Hepatic Dysfunction^b^	Yes	6 (0.6%)	12 (2.5%)	<.001*
		No	1,018 (99%)	465 (97%)	
	Renal Dysfunction^b^	Yes	45 (4.4%)	32 (6.7%)	0.058
		No	979 (96%)	445 (93%)	
	Multi-Organ Dysfunction (MODS)^b^	Yes	35 (3.4%)	78 (16%)	<.001*
		No	989 (97%)	399 (84%)	
	Disease-Related Emergency^b^	Hyperleukocytosis	6 (0.6%)	9 (1.9%)	<.001*
		SMS	16 (1.6%)	7 (1.5%)	
		TLS	109 (11%)	12 (2.5%)	
		No Emergency	893 (87%)	449 (94%)	
ICU Interventions	Renal Replacement Therapy (RRT)^b^	Yes	70 (6.8%)	38 (8.0%)	0.400
		No	954 (93%)	439 (92%)	
	Mechanical Ventilation^b^	Yes	253 (25%)	464 (97%)	<.001*
		No	771 (75%)	13 (2.7%)	
	Inotropic Support^b^	Yes	189 (18%)	410 (86%)	<.001*
		No	835 (82%)	67 (14%)	

ICU, intensive care unit; IQR, interquartile range; Data are *n* (%) or median [IQR]. The calculated *p*-values derived from the Wilcoxon rank-sum test for continuous variables.

^a^ Pearson's Chi-squared test or Fisher's exact test for categorical variables.

^b^ As appropriate.

* Significant at *P* ≤ 0.05.

** Significant at *P* ≤ 0.01.

The multivariable logistic regression analysis identified factors independently associated with ICU mortality after adjusting for other variables.

Several demographic and clinical characteristics were identified as significant predictors. Younger age was associated with higher odds of mortality, with each additional year of age decreasing the odds by 4% [OR = 0.96, 95% CI (0.94, 0.99), *p* = .002]. Both the longer length of hospital stay prior to ICU admission [LOS Inpatient; OR = 1.01 per day, 95% CI (1.00, 1.01), *p* = .046] and longer length of stay within the ICU [LOS ICU; OR = 1.04 per day, 95% CI (1.03, 1.06), *p* < .001] were associated with incrementally increased odds of mortality.

Admission circumstances also played a significant role. Patients transferred from an inpatient ward (IP) had significantly higher odds of death compared to those admitted from the emergency room (ER) [OR = 1.63, 95% CI (1.20, 2.22), *p* = .002]. Furthermore, patients with progressive disease at the time of ICU admission faced significantly higher odds of mortality compared to those whose disease was not progressive [OR = 1.86, 95% CI (1.38, 2.50), *p* < .001].

The presence of specific organ dysfunctions at ICU admission demonstrated strong independent associations with mortality. The odds of death were substantially increased for patients experiencing respiratory failure [OR = 6.35, 95% CI (4.13, 9.97), *p* < .001], septic shock [OR = 6.01, 95% CI (3.97, 9.23), *p* < .001], hepatic dysfunction [OR = 6.01, 95% CI (1.88, 20.70), *p* = .003], or renal dysfunction [OR = 5.43, 95% CI (2.95, 10.00), *p* < .001], compared to those without these conditions. Cerebral failure [OR = 2.96, 95% CI (1.86, 4.77), *p* < .001] and cardiovascular (CVS) dysfunction [OR = 2.91, 95% CI (1.91, 4.49), *p* < .001] were also independently associated with significantly increased odds of ICU mortality (Table 3).

**Table 3:** Independent predictors of ICU mortality from multivariable logistic regression analysis.

Predictors	OR	95% CI	*p*-value
Age	0.96	0.94, 0.99	0.002**
LOS Inpatient	1.01	1.00, 1.01	0.046*
LOS ICU	1.04	1.03, 1.06	<.001**
Transfer from: IP (ref = ER)	1.63	1.20, 2.22	0.002**
Disease status: Progressive (ref = Not progressive)	1.86	1.38, 2.50	<.001**
Septic shock: Yes (ref = No)	6.01	3.97, 9.23	<.001**
CVS dysfunction: Yes (ref = No)	2.91	1.91, 4.49	<.001**
Respiratory failure: Yes (ref = No)	6.35	4.13, 9.97	<.001**
Cerebral failure: Yes (ref = No)	2.96	1.86, 4.77	<.001**
Hepatic dysfunction: Yes (ref = No)	6.01	1.88, 20.70	0.003**
Renal dysfunction: Yes (ref = No)	5.43	2.95, 10.00	<.001**

OR, odds ratio; CI, confidence interval. Ref represents the reference category.

* Significant at *P* ≤ 0.05.

** Significant at *P* ≤ 0.01.

**Figure 1:**
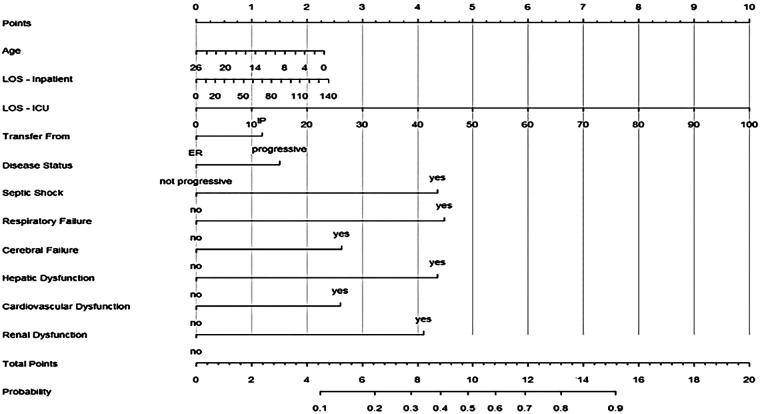
Nomogram for predicting ICU mortality in pediatric oncology patients.

Moreover, a nomogram developed from the multivariable logistic regression model to predict the probability of mortality for individual pediatric oncology patients at ICU admission is presented in Figure 1. The nomogram incorporates the significant predictors identified in the multivariable model. It includes age, length of hospital stay prior to ICU (LOS-Inpatient), length of ICU stay (LOS-ICU), transfer source (Inpatient vs. ER), disease status (Progressive vs. Not Progressive), and the presence of septic shock, respiratory failure, cerebral failure, hepatic dysfunction, cardiovascular dysfunction, and renal dysfunction.

The nomogram assigns points to each predictor based on its adjusted contribution to mortality risk; higher-risk factors, such as younger age, inpatient transfer, progressive disease, specific organ failures, and septic shock receive more points. Summing the points across all factors yields a total score, which corresponds to an estimated probability of ICU mortality on the bottom scale. This graphical tool offers a user-friendly way to visualize mortality risk and may aid in decision-making, such as prompting earlier or more aggressive interventions for patients with higher scores.

Regarding the primary attributed causes of death among the 477 pediatric oncology patients who died during their ICU stay, septic shock was the most frequent cause, responsible for nearly half of all deaths (*n* = 220, 46%). Disease Progression/Relapse was the second leading cause, accounting for 28.6% of deaths (*n* = 136). Treatment-related mortality (TRM) was the third most common cause (*n* = 121, 25.4%) (Table 4). Treatment-related mortalities (TRM) included conditions such as acute heart failure, respiratory failure (including pulmonary hemorrhage, ARDS, radiation pneumonitis, and advanced lung graft-vs.-host disease), as well as severe veno-occlusive disease (VOD).

**Table 4:** Primary attributed causes of death among pediatric oncology ICU non-survivors.

Cause of Death	*n*	%
Septic Shock	220	46
Disease Progression/Relapse	136	28.6
Treatment-Related Mortality (TRM)	121	25.4
Total Deaths	477	100

## Discussion

Children undergoing cancer treatment are more prone to critical illness due to their underlying disease, immunosuppression, and treatment-related toxicities (11). Many of these patients, estimated at up to 40% over their illness course, will be admitted to a Pediatric Intensive Care Unit (PICU) (5). Although survival rates for pediatric cancer have greatly increased in high-income countries (HICs), outcomes after PICU admission, especially in low- and middle-income countries (LMICs), continue to be a major problem (8, 12). Research shows that death rates for pediatric oncology patients needing intensive care are much higher than the general PICU population and often trail behind advances shown in adult oncology critical care or total pediatric cancer survival (5, 13, 14). Moreover, data particular to LMICs show significant variation and often worse results than HICs, hence underlining the influence of resource constraints and system-level elements (8, 12, 15, 16).

Aiming to identify the mortality predictors for pediatric cancer patients admitted to our ICU, our research was done at the Children's Cancer Hospital Egypt 57357 (CCHE-57357), a major tertiary pediatric oncology facility in an LMIC. This study aimed to identify local risk factors to guide treatments and improve outcomes in our setting. Among 1501 ICU admissions at CCHE-57357 between 2019 and 2022, our study found an overall death rate of 32%. This finding is somewhat above the pooled LMIC estimate of 30.3% in a recent meta-analysis by Gabela et al. (12). Though this rises to around 33%–35% when removing lower-risk post-operative patients, it is higher than the death rates reported from well-resourced HICs, where the pooled rate is about 28% (5). However, our 32% rate within the LMIC environment itself is an improvement above the 40% mortality reported a few years earlier by the South Egypt Cancer Institute (8). This might indicate progress over time, variations in patient demographics, or even the advantages of CCHE-57357's specialized infrastructure and committed pediatric oncology emphasis. Common to LMICs, such as limited resources, possible delays in referral and presentation (8, 9), greater malnutrition or coinfection burden, and perhaps higher rates of antimicrobial resistance (17), likely play a major role in mortality in both general and specialized units.

Our multivariable study found many independent variables strongly linked to ICU death. Firstly, older age was linked to somewhat decreased chances of death (OR 0.96, *p* = .002), so younger children had a greater risk. Our findings correspond with the natural vulnerability of infants and very young children undergoing cancer treatment. Younger children tend to have less physiological reserve, which makes them more prone to fast decline (17). This younger age range enriches for particular high-risk pediatric malignancies such as infantile ALL or AML.

In addition, increased death was independently linked to both longer hospital stay before ICU admission (LOS Inpatient) and longer length of ICU stay itself (LOS ICU) (OR 1.01 and 1.04 per day, respectively). A longer pre-ICU stay may indicate a delay in identifying the initial signs of deterioration, as well as the added risks of prolonged hospital admission (8). A longer ICU stay, although required for certain survivors, is also a sign of sickness severity, extended organ support requirements, and a higher chance of developing ICU-related problems, including infections, which increase the likelihood of a poorer outcome (5, 17).

Moreover, patients moved from an inpatient ward (IP) had much higher death rates than those admitted straight from the Emergency Room (ER) (OR 1.63, *p* = .002). The comparison between outpatient vs. inpatient may reflect the issues related to delayed identification of decline, as discussed by Ali et al. (8). This highlights the necessity of implementing an early warning score system (PEWS) to facilitate timely referrals to the ICU before significant and irreversible clinical deterioration has occurred. Implementation of pediatric early warning signs scoring systems in 32 resource-limited hospitals in Latin America has shown reduced clinical deterioration event mortality in pediatric patients, as reported by Asya Agulnik et al. (18). At the time of data collection for this study, PEWS was not implemented at our center. A formal PEWS process at our hospital was instituted in 2023 and an analysis of the impact of this program is being developed. Moreover, admission from an inpatient setting could indicate deterioration related to hospital-acquired infections, initial treatment failures, or a cohort that is naturally sicker and has not responded to ward-level support, compared to acutely presenting emergencies.

Progressive disease/Relapse at the time of ICU admission was a robust predictor of death compared to non-progressive disease (OR 1.86, *p* < .001). This is consistent with results by Ali et al. (8) who found worse outcomes for patients hospitalized during relapse or progression relative to those at first diagnosis or receiving treatment in remission. It also aligns with the results from Azevedo et al. (19), which identified cancer recurrence as a predictor of in-hospital death, regardless of severity, in children with malignancies and septic shock admitted to the ICU at a tertiary hospital in Brazil.

In our cohort, the presence of cardiovascular dysfunction, septic shock, respiratory failure, hepatic dysfunction, renal dysfunction, and cerebral failure at ICU admission were all strong independent predictors of increased ICU mortality, with especially high odds ratios for respiratory failure (OR 6.35), septic shock (OR 6.01), hepatic dysfunction (OR 6.01), and renal dysfunction (OR 5.43). These findings are highly consistent with several previous studies. For example, Ali et al. (8) in Egypt noted that admission for systemic infection (usually resulting in septic shock) and respiratory failure had the lowest survival rates (52.6% and 47.8% respectively). Furthermore, the number of failing organs was a strong predictor, with mortality approaching 85% in patients with multi-organ failure. Key mortality predictors identified in an Ethiopian prospective study by Bacha et al. (9) as well as the meta-analysis by Molla et al. (20) were sepsis, poor GCS (suggesting brain dysfunction), MODS, and the requirement for inotropes (suggesting cardiovascular dysfunction).

Septic shock was the most attributable cause of death in our group, consistent with the established predictors, comprising 46% of deaths. Disease progression/Relapse (28.6%) came next, followed by Treatment-related mortality (TRM, 25.4%). The predominance of septic shock as the main cause of mortality is consistent with several studies from LMICs (8, 9, 17) and also occurs in HIC reports (5). Neutropenia, immunosuppressive treatments, indwelling central lines, and mucositis make children with cancer more vulnerable to serious infections and sepsis (8, 16). In LMIC environments, several factors, including inadequate hygiene, malnutrition, late presentation, restricted diagnostic capacity, and antibiotic resistance, might increase the risk and severity of sepsis even more (9, 17, 20). The notable role of TRM and disease progression further underlines the natural hazards of aggressive cancer treatments and the difficulties experienced when the underlying illness progresses even with therapy.

### Strengths and limitations

This study has many important findings. First, it represents one of the largest cohorts for pediatric oncology patients, admitted to a PICU in a low and middle-income country. It provides data from more than 1500 ICU admissions in CCHE-57357, a dedicated pediatric academic cancer center. This large sample size improves statistical power to identify important predictors of mortality. In addition, the use of multimodal logistics regression analysis enables the identification of independent predictions when adjusting for potential configurations, and the latter development of a nomogram provides a practical tool for risk stratification. The study provides valuable local data that can inform the initiative for quality improvement.

However, this study also has many limitations that should be considered when interpreting the conclusions. The retrospective cohort design, while allowing for the analysis of a large dataset, is inherently susceptible to information bias due to reliance on existing medical records, which may contain incomplete or inconsistent data. Unmeasured confounding variables, such as specific socioeconomic factors, detailed variations in treatment protocols not captured, or precise timing of all interventions, may have influenced the observed associations. As a single-center study, conclusions, even though they are important for CCHE-57357, can be limited to other PICUs in Egypt or other LMICs, where individual patient populations, resource preparation, or standard operating processes differ.

## Conclusion

Among pediatric oncology patients admitted to the PICU at CCHE-57357 in an LMIC environment, this study investigated mortality predictors and found a 32% mortality rate. Key independent predictors included younger age, transfer from an inpatient setting, progressive disease/relapse, and organ dysfunctions at the time of ICU admission, especially septic shock and respiratory failure. These results underline the need for focused quality improvement projects aimed at early identification and aggressive management of sepsis and respiratory failure, optimized care pathways for deteriorating inpatients, and customized supportive care strategies for high-risk populations such as younger children and those with advanced disease, implying possibilities for enhancing outcomes in this vulnerable group.

## Data Availability

The original contributions presented in the study are included in the article/Supplementary Material, further inquiries can be directed to the corresponding author.
